# Intracorporeal versus extracorporeal anastomosis for minimally invasive right colectomy: A multi-center propensity score-matched comparison of outcomes

**DOI:** 10.1371/journal.pone.0206277

**Published:** 2018-10-24

**Authors:** Robert K. Cleary, Andrew Kassir, Craig S. Johnson, Amir L. Bastawrous, Mark K. Soliman, Daryl S. Marx, Luca Giordano, Tobi J. Reidy, Eduardo Parra-Davila, Vincent J. Obias, Joseph C. Carmichael, Darren Pollock, Alessio Pigazzi

**Affiliations:** 1 Department of Surgery, Division of Colon and Rectal Surgery, St Joseph Mercy Hospital, Ann Arbor, Michigan, United States of America; 2 Colon and Rectal Clinic of Scottsdale, Scottsdale, Arizona, United States of America; 3 Department of Surgery, Oklahoma Surgical Hospital, Tulsa, Oklahoma, United States of America; 4 Swedish Colon and Rectal Clinic, Division of Colon and Rectal Surgery, Swedish Medical Center, Seattle, Washington, United States of America; 5 Colon and Rectal Clinic of Orlando, Orlando, Florida, United States of America; 6 Department of Surgery, Monroe Surgical Hospital, Monroe, Louisiana, United States of America; 7 Division of Gastrointestinal and Colorectal Surgery, Minimally Invasive and Robotic-assisted Surgery, and Bariatric Surgery, Jefferson Health Northeast Torresdale, Philadelphia, Pennsylvania, United States of America; 8 Department of Surgery, St. Francis Hospital and Health Centers, Franciscan Alliance, Indianapolis, Indiana, United States of America; 9 Department of Surgery, Celebration Center for Surgery, Florida Hospital Medical Group, Celebration, Florida, United States of America; 10 Division of Colon and Rectal Surgery, George Washington University, Washington, District of Columbia, United States of America; 11 Department of Surgery, Division of Colon and Rectal Surgery, University of California Irvine, Irvine, California, United States of America; 12 Swedish Colon and Rectal Clinic, Division of Colon and Rectal Surgery, Swedish Medical Center, Seattle, Washington, United States of America; 13 Department of Surgery, Division of Colon and Rectal Surgery, University of California Irvine, Irvine, California, United States of America; Royal Prince Alfred Hospital, AUSTRALIA

## Abstract

**Background:**

The primary objective of this study was to retrospectively compare short-term outcomes of intracorporeal versus extracorporeal anastomosis for minimally invasive laparoscopic and robotic-assisted right colectomies for benign and malignant disease. Recent studies suggest potential short-term outcomes advantages for the intracorporeal anastomosis technique.

**Methods:**

This is a multicenter retrospective propensity score-matched comparison of intracorporeal and extracorporeal anastomosis techniques for laparoscopic and robotic-assisted right colectomy between January 11, 2010, and July 21, 2016.

**Results:**

After propensity score-matching, there were a total of 1029 minimal invasive surgery cases for analysis—379 right colectomies (335 robotic-assisted and 44 laparoscopic) done with an intracorporeal anastomosis and 650 right colectomies (253 robotic-assisted and 397 laparoscopic) done with an extracorporeal anastomosis. There were no significant differences in any preoperative patient characteristics between groups. The minimally invasive intracorporeal anastomosis group had significantly longer operative times (p<0.0001), lower conversion to open rate (p = 0.01), shorter hospital length of stay (p = 0.02) and lower complication rate from after discharge to 30-days (p = 0.04) than the extracorporeal anastomosis group.

**Conclusions:**

This comparison shows several clinical outcomes advantages for the intracorporeal anastomosis technique in minimally invasive right colectomy. These data may guide future refinements in minimally invasive training techniques and help surgeons choose among different minimally invasive options.

## Introduction

Several studies have demonstrated short-term outcomes advantages for the minimally invasive surgery (MIS) approach when compared to open colorectal surgery.[1–8] Even with these advantages, only 50–60% of colectomies and 10–20% of rectal resections are completed with a MIS approach.[9–13]

When considering MIS options, there may be clinical outcomes advantages to the intracorporeal approach when compared to the extracorporeal technique.[14–17] For MIS right colectomy, the extracorporeal anastomosis is typically performed after delivering the specimen through a midline incision extraction site that may result in traction injury to the ileum and colon, and an increased rate of extraction site hernia.[16, 18–20] In contrast, the intracorporeal anastomosis technique is conducted after the specimen is completely detached from surrounding structures and allows the specimen to be removed from extraction site incisions less prone to incisional hernia.[15–17, 21–25]

The purpose of this multi-institutional retrospective study was to compare the short-term clinical outcomes of right colectomy MIS (laparoscopic or robotic-assisted) performed with intracorporeal versus extracorporeal anastomosis for benign and malignant disease.

## Methods

### Data source

This study is multi-institutional and retrospective. De-identified peri-operative information for consecutive MIS cases were collected from existing medical records of patients who underwent laparoscopic and robotic-assisted right colectomies for benign and malignant disease at 11 participating institutions in the United States between January 11, 2010, and July 21, 2016. The study was conducted in accordance with institutional review board guidelines at each institution. Data were retrieved using standardized data collection forms to ensure uniformity across participating sites. Operative approach data for colorectal operations included identification of type of anastomosis and converted cases through detailed surveillance of the operative report dictated by the surgeon. Study-specific informed consent waivers for retrospective data collection and Institutional Review Board approval were obtained from each participating clinical site. Clinical site Institutional Review Boards (IRB) included Saint Joseph Mercy Health System IRB, ARIA Health IRB, Western IRB, Honor Health IRB, Florida Hospital IRB, University of California, Irvine, IRB, Orlando Regional Medical Center IRB.

All study patient information was kept confidential and managed in compliance with Health Insurance Portability and Accountability Act of 1996 requirements. The study chairs (RKC and AP) recruited surgeons based on reputation for operative approaches and attendance at national meetings. Surgeons at participating sites were reviewed for eligibility per study protocol and had performed a minimum of 50 laparoscopic and/or robotic-assisted right colectomy cases prior to contributing to a study arm. Most surgeons contributed MIS cases to both the intra- and extracorporeal study arm based on their current practice for the treatment of benign and malignant right colon disease. One surgeon contributed MIS cases to the extracorporeal and not the intracorporeal study arm.

### Study cohort

Eligible patients were ≥ 18 years of age and underwent an elective MIS (robotic-assisted or laparoscopic) right colectomy with intracorporeal or extracorporeal anastomosis either at or proximal to the mid-transverse colon for benign or malignant neoplasia, or inflammatory bowel disease. Exclusion criteria were perforated, obstructing, or locally invasive neoplasms; emergency procedures; patients undergoing right colectomy as a secondary procedure; patients receiving neoadjuvant or postoperative radiation therapy for malignant neoplasia.

For the comparison of intra-and extracorporeal anastomosis techniques, baseline patient characteristics as well as postoperative short term clinical and pathologic outcomes were obtained from hospital medical records.

### Surgical technique

Eligible patients underwent elective right colectomy commensurate with the dissection technique of the individual surgeon up to the mid transverse colon for benign or malignant neoplasia and inflammatory bowel disease using a robotic-assisted approach with intracorporeal or extracorporeal anastomosis, or using a laparoscopic approach with intracorporeal or extracorporeal anastomosis.

#### Extracorporeal anastomosis technique

Pneumoperitoneum, port placement, and robot docking (for the robotic-assisted group) were achieved after induction of general anesthesia by the operating surgeon method of choice. Medial to lateral and/or lateral to medial dissection was performed and the ileocolic vessels were identified. The extent of intracorporeal or extracorporeal vessel ligation and division, and the degree of intracorporeal or extracorporeal mesenteric dissection were determined by surgeon preference. The gastrocolic ligament was taken down and the hepatic flexure was mobilized. After mobilization of the right colon, a midline port incision—typically the camera port incision—was extended to serve as the extraction site. The specimen was delivered through the midline extraction incision and the anastomosis conducted using standard open techniques. The use of wound protectors and closure methods were per surgeon preference.

#### Intracorporeal anastomosis technique

Pneumoperitoneum, port placement, and robot docking (for the robotic-assisted group) were performed after the induction of general anesthesia per surgeon preference. The entire operation including the anastomosis was done by intracorporeal techniques. Medial-to-lateral and lateral-to-medial dissection, ligation and division of the ileocolic, right colic, and middle colic branches were all systematically performed. The right mesocolon was taken down from point of transection of the ileocolic vessels to the terminal ileum and to a pre-determined point on the transverse colon. The transverse colon and ileum were then divided with a robotic-assisted or laparoscopic stapler. The terminal ileum was aligned with the transverse colon in either an isoperistaltic or antiperistaltic configuration with attention turned to constructing the anastomosis. A colotomy and enterotomy were created to form a common enterotomy channel. After performing a stapled anastomosis, the common enterotomy was closed with suturing or stapling techniques. The specimen was extracted through an off-midline (muscle splitting transverse) or Pfannenstiel incision. The use of wound protectors and closure methods were per surgeon preference.

All robotic-assisted right colectomies were performed with either the *da Vinci Si* or *da Vinci Xi* Surgical System (Intuitive Surgical, Inc.). For both intracorporeal and extracorporeal techniques, no attempt was made to control for utilization of the da Vinci Si or da Vinci Xi robotic-assisted systems, or for total mesocolic excision with central vascular ligation.

### Outcome and explanatory variables

Baseline data collected for the statistical model included patient demographics (age, gender), general health factors (BMI, American Society of Anesthesiologists (ASA) Class, functional health status), patient comorbidities (hypertension, diabetes mellitus, cardiopulmonary disorders, vascular disorders, bleeding disorders, gastrointestinal disorders, genitourinary disorders), previous abdominal surgery, preoperative diagnosis (malignant neoplasia, benign neoplasia, inflammatory bowel disease), and pathologic characteristics (tumor size, tumor stage, tumor location).

The outcomes retrieved for comparison of intra- and extracorporeal anastomosis technique study groups included intraoperative data (operative time, conversion to open laparotomy, estimated blood loss), recovery outcomes (days to first bowel movement, hospital length of stay, need for transfusions), total intraoperative procedure-related complications (bleeding requiring additional intervention, visceral injury: bladder, spleen, gastric, small bowel, vascular injury requiring additional intervention), complications related to anesthesia, anastomotic complications), and total postoperative procedure-related complications (surgical site infections (SSI), other wound complications, gastrointestinal complications, postoperative bleeding with or without transfusion, deep venous thrombosis (DVT), genitourinary complications, cardiac complications, pulmonary complications, renal failure) diagnosed both during the index hospitalization prior to discharge as well as after discharge up to 30 days.

### Statistical analysis

All patient characteristics and study outcomes were summarized in terms of rates, means, standard deviations, and percentages as appropriate. Comparisons for categorical variables were made using a Pearson Chi-squared test or Fisher’s exact test as appropriate. Continuous variables were compared using a Student’s t-test or an independent samples t-test with Satterthwaite approximation. All analyses were based on available data. No missing data imputation was performed.

Because subjects were not randomly assigned to treatment arms, propensity score matching (PSM) was applied to obtain an approximate unbiased measure of outcomes by balancing important demographics and preoperative characteristics. Matching was performed using a multivariable logistic model to determine the probability of undergoing MIS with an intracorporeal anastomosis. A quintile stratification matching algorithm was applied. The covariates include BMI, ASA Class, comorbidities (hypertension, diabetes mellitus, cardiopulmonary disorders, vascular disorders, bleeding disorders, gastrointestinal disorders, genitourinary disorders), preoperative diagnosis, and previous abdominal surgery (exploratory/diagnostic procedures). Each Propensity Score Model was evaluated according to Faries *et al*., (2010)[26] to ensure best propensity score distribution and best overall PSM fit. The adjusted analysis of all binary and continuous outcomes was carried out using a regression model stratified on propensity score quintile. In case of binary outcome, logistic regression was employed. Ordinary outcomes were evaluated with van Elteren test for stratified ordinary data. Statistical significance was defined as p<0.05.

All analyses were carried out using SAS 9.4 software for Windows (SAS Institute, Inc., Cary, NC, USA).

## Results and discussion

**Fig 1:**
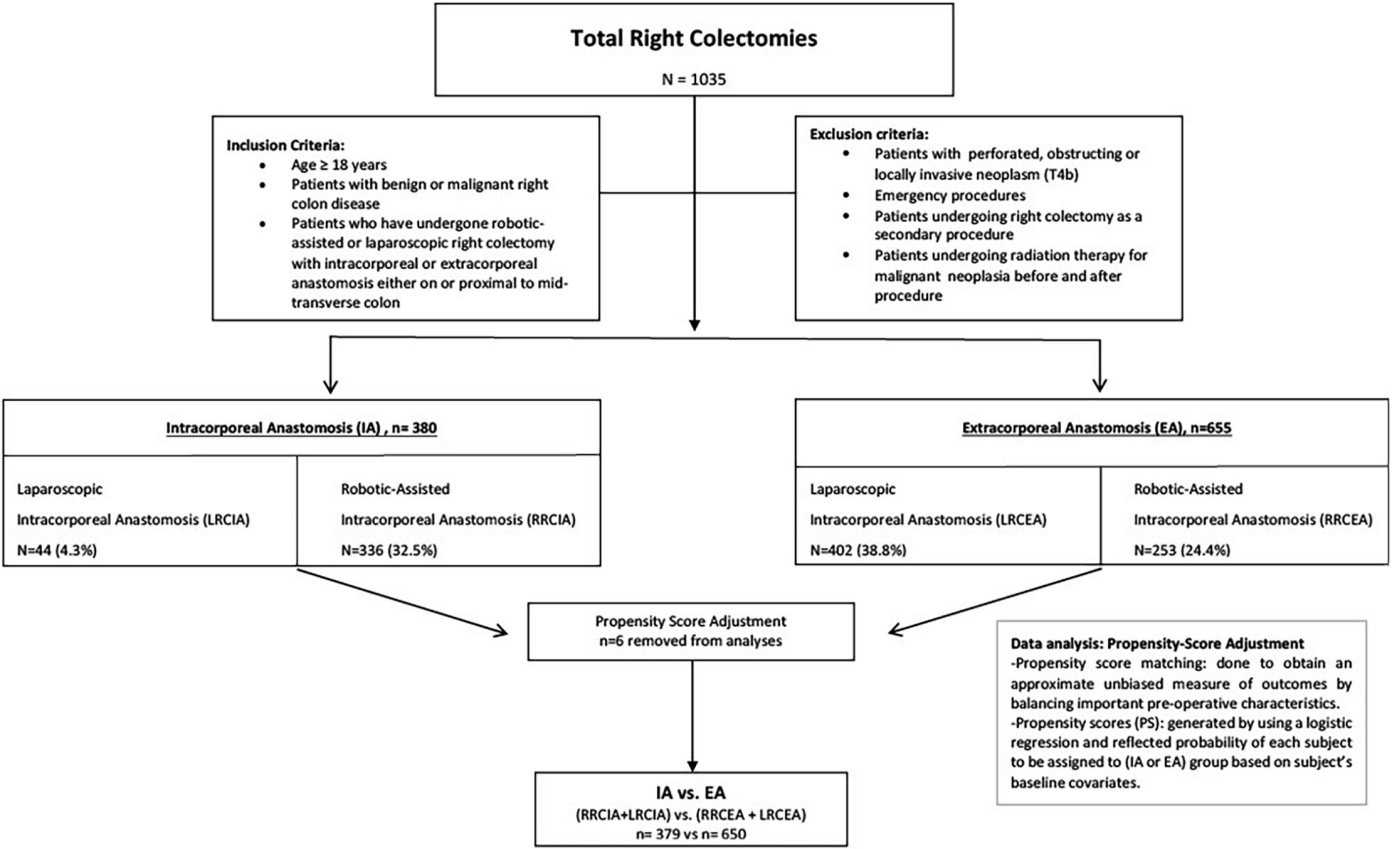
Patient distribution in treatment arms.

Fig 1 demonstrates the total number of study patients and the number of patients in each study group before and after propensity score adjustment. Recruiting surgeons for the laparoscopic right colectomy intracorporeal (LRCIA) group was challenging because of the small number of surgeons using this technique. The number of patients in the LRCIA study arm was therefore considerably smaller than the other study arms. Propensity scores were not assigned to 6 patients because of unknown baseline medical history covariates (comorbidities and previous abdominal surgery)—one patient in the robotic right colectomy intracorporeal (RRCIA) group and 5 patients in the laparoscopic right colectomy extracorporeal (LRCEA) group. Patient demographics and preoperative characteristics were statistically similar across both groups regarding age, gender, BMI, ASA group, previous abdominal surgery, preoperative diagnosis and comorbidity (Table 1).

**Table 1:** Patient demographics and preoperative characteristics of intracorporeal and extracorporeal groups.

Variables	IA n = 379 (RRCIA n = 335 LRCIA n = 44)	EA n = 650 (RRCEA n = 253 LRCEA n = 397)	Unadjusted p-value^a^	Adjusted p-value^b^
Age (years)				
Mean ± SD [n]	66.2 ± 12.1 [379]	65.9 ± 13.3 [650]	0.71	0.83
Gender, n (%)				
Female	199 (52.5%)	316 (48.6%)	0.23	0.16
Male	180 (47.5%)	334 (51.4%)		
BMI				
Mean ± SD [n] (95% CI)	29.6 ± 6.5 [379] (28.9, 30.2)	28.2 ± 6.1 [650] (27.8, 28.7)	0.001	0.84
ASA group				
ASA class 1–2	167 (44.1%)	380 (58.5%)	<0.0001	0.62
ASA class 3–6	208 (54.9%)	258 (39.7%)		
Unknown**	4 (1.1%)	12 (1.8%)		
Previous Abdominal Surgery, n (%)	196 (51.7%)	309 (47.5%)	0.34	0.33
Preoperative Diagnosis, n (%)				
Benign Neoplasm	180 (47.5%)	302 (46.5%)	0.75	0.84
Malignant Neoplasm	178 (47.0%)	298 (45.8%)	0.73	0.82
Inflammatory Bowel Disease	18 (4.7%)	43 (6.6%)	0.22	0.96
≥ 1 Comorbidity, n (%)	288 (76.0%)	468 (72.0%)	0.16	0.99

IA = Intracorporeal, EA = Extracorporeal, SD = standard deviation, min = minutes, n = number

RRCIA = Robotic Right Colectomy Intracorporeal Anastomosis, LRCIA = Laparoscopic Right Colectomy Intracorporeal Anastomosis

RRCEA = Robotic Right Colectomy Extracorporeal Anastomosis, LRCEA = Laparoscopic Right Colectomy Extracorporeal Anastomosis

** = unknown or missing data

^a^p value before propensity score adjustment

^b^p value after propensity score adjustment

There were no statistically significant differences in the post-operative pathological outcomes between the intracorporeal and extracorporeal anastomosis groups. One hundred eighty-seven (49.3%) in the IA group and 307 (47.2%) in the EA group had malignant neoplasia (p = 0.99). The mean tumor size was similar in the IA and EA groups (mean ±SD: 4.1 ±2.1 cm and 4.3 ±2.5 cm respectively (p = 0.45). There was no significant difference in pathologic tumor stage (p = 0.57), and tumor location defined as cecum, ascending colon, hepatic flexure, and up to mid transverse colon (p = 0.68) between the two groups (Table 2).

**Table 2:** Postoperative pathology.

Postoperative Pathology	IA n = 187	EA n = 307	Adjusted p-value^b^
Malignant Yes, N (%)	187 (49.3%)	307 (47.2%)	0.99
Size (cm)			
Mean ± SD [n] (95% CI)	4.1 ± 2.1 [185] (3.8, 4.4)	4.3 ± 2.5 [307] (4.0, 4.5)	0.45
Median (95% CI)	4.0 (3.4, 4.0)	4.0 (4.0, 4.0)	
Pathological Stage^1^, N (%)			
Stage 0	9 (4.8%)	10 (3.3%)	0.57
Stage I	36 (19.3%)	80 (26.1%)	
Stage II	63 (33.7%)	104 (33.9%)	
Stage III	69 (36.9%)	86 (28.0%)	
Stage IV	8 (4.3%)	25 (8.1%)	
Unknown**	2 (1.1%)	2 (0.7%)	
Tumor Location^1^, N (%)			
Cecum	79 (42.2%)	131 (42.7%)	0.68
Ascending Colon	90 (48.1%)	127 (41.4%)	
Hepatic Flexure	7 (3.7%)	16 (5.2%)	
Transverse Colon	10 (5.3%)	28 (9.1%)	
Unknown**	1 (0.5%)	5 (1.6%)	

IA = Intracorporeal, EA = Extracorporeal, SD = standard deviation, cm = centimeter, CI = confidence interval n = number

^1^Combined other and unknown to one group called Unknown**

^b^p value after propensity score adjustment

The IA group had a significantly lower conversion rate (p = 0.01), less estimated blood loss (p = 0.001), shorter time to first bowel movement (p = 0.01), and shorter hospital LOS (p = 0.02) than the EA group. There was only one (0.3%) conversion in the IA group and the reason was advanced cancer. The 19 (2.9%) conversions in the EA group were for intra-abdominal adhesions (n = 10), advanced cancer (n = 1), morbidly obese patients (n = 2), patient not suitable for minimally invasive approach (n = 1), bulky mass (n = 1), intraoperative bleeding (n = 1), disease characteristics (n = 2), and technical challenge of maintaining insufflation (n = 1). The OR time was significantly longer in the IA group (mean ±SD: 3.1 ± 0.9 hours) than in the EA group (mean ±SD: 2.5 ± 1.0 hours) (p<0.0001) (Table 3).

**Table 3:** Procedure-related outcomes and overall complication rates.

Variables	IA n = 379 (RRCIA n = 335 LRCIA n = 44)	EA n = 650 (RRCEA n = 253 LRCEA n = 397)	Adjusted p-value^b^
Conversion to open surgery, n (%)	1 (0.3%)	19 (2.9%)	0.01
Estimated blood loss (ml), Mean +/-SD [n]	58.9 ± 84.0 [378]	77.5 ± 102.8 [638]	0.001
Transfusion, Yes, n (%)	4 (1.1%)	10 (1.5%)	0.42
OR time (Wheels in/out) (hours), Mean +/-SD [n]	3.1 ± 0.9 [320]	2.5 ± 1.0 [574]	<0.0001
Days to first bowel movement			
Mean ± SD [n]	2.7 ± 1.5 [315]	2.9 ± 1.5 [571]	0.01
Median (95% CI)	3 (2, 3)	3 (3, 3)	
Hospital LOS (days)			
Mean ± SD [n] (95% CI)	4.0 ± 2.8 [379] (3.7, 4.3)	4.5 ± 3.4 [649] (4.2, 4.7)	0.02
Median (95% CI)	3 (3, 4)	4 (4, 4)	
Intra-operative complications, n (%)	2 (0.5%)	3 (0.5%)	0.99
Post-operative complications prior to discharge, n (%)	57 (15.0%)	88 (13.5%)	0.55
Post-operative complications hospital discharge to 30 days n (%)	18 (5.0%)	53 (8.9%)	0.04

IA = Intracorporeal, EA = Extracorporeal

RRCIA = Robotic Right Colectomy Intracorporeal Anastomosis, LRCIA = Laparoscopic Right Colectomy Intracorporeal Anastomosis

RRCEA = Robotic Right Colectomy Extracorporeal Anastomosis, LRCEA = Laparoscopic Right Colectomy Extracorporeal Anastomosis

SD = standard deviation, min = minutes, ml = milliliters, n = number

^b^p value after propensity score adjustment

There was no significant difference in intraoperative complication rates (p = 0.99) and postoperative complication rates during the index hospitalization prior to discharge (p = 0.55) between the two groups. The overall complication rate from index hospitalization discharge up to 30 days after discharge was significantly lower in the intracorporeal anastomosis group than in the extracorporeal anastomosis group (5.0% vs. 8.9% respectively, p = 0.04) (Table 3).

Specific complication rates were reported separately. *Clostridium* difficile colitis was slightly more prevalent in the extracorporeal group (Table 4). Postoperative complication rates were similar between IA and EA groups with respect to cardiovascular, pulmonary, anesthetic, gastrointestinal, and genitourinary complications, surgical site infections, paralytic ileus, anastomotic leaks, and bleeding complications (Table 4).

**Table 4:** Complications during index hospitalization prior to discharge.

Postoperative Complications, prior to discharge, n %	IA	EA
	n = 379 (RRCIA n = 335 LRCIA n = 44)	n = 650 (RRCEA n = 253 LRCEA n = 397)
Cardiovascular Complications	9 (2.4%)	11 (1.7%)
Gastrointestinal Complications	0 (0.0%)	6 (0.9%)
Genitourinary Complications	8 (2.1%)	13 (2.0%)
Pulmonary Complications	10 (2.6%)	11 (1.7%)
Anesthetic Complications	2 (0.5%)	1 (0.2%)
Post-operative Bleeding	16 (4.2%)	29 (4.5%)
Surgical Site Infection	2 (0.5%)	9 (1.4%)
Anastomotic Leakage	0 (0.0%)	6 (0.9%)
Paralytic Ileus	9 (2.4%)	19 (2.9%)
Small Bowel Obstruction	1 (0.3%)	5 (0.8%)
*Clostridium* difficile Colitis	0 (0.0%)	6 (0.9%)

IA = Intracorporeal, EA = Extracorporeal

RRCIA = Robotic Right Colectomy Intracorporeal Anastomosis, LRCIA = Laparoscopic Right Colectomy Intracorporeal Anastomosis

RRCEA = Robotic Right Colectomy Extracorporeal Anastomosis, LRCEA = Laparoscopic Right Colectomy Extracorporeal Anastomosis

Gastrointestinal complications and surgical site infections following discharge up to 30-days were also higher in the extracorporeal group than the intracorporeal anastomosis group (Table 5).

**Table 5:** Complications from discharge up to 30 days.

Postoperative Complications from Discharge to 30 Days	IA	EA
	N = 379 (RRCIA n = 335 LRCIA n = 44)	N = 650 (RRCEA n = 253 LRCIA n = 397)
Cardiovascular Complications	0 (0.0%)	0 (0.0%)
Gastrointestinal Complications	2 (0.6%)	10 (1.7%)
Genitourinary Complications	1 (0.3%)	4 (0.7%)
Pulmonary Complications	0 (0.0%)	3 (0.5%)
Post-operative Bleeding	4 (1.1%)	6 (1.0%)
Surgical Site Infection	5 (1.4%)	16 (2.7%)
Other Wound Complications	0 (0.0%)	2 (0.3%)
Paralytic Ileus	2 (0.6%)	5 (0.8%)
Small Bowel Obstruction	0 (0.0%)	2 (0.3%)
Bowel Obstruction	0 (0.0%)	1 (0.2%)
C. difficile Colitis	1 (0.3%)	1 (0.2%)
Other	3 (0.8%)	2 (0.3%)

IA = Intracorporeal, EA = Extracorporeal, SD = standard deviation, min = minutes, n = number

RRCIA = Robotic Right Colectomy Intracorporeal Anastomosis, LRCIA = Laparoscopic Right Colectomy Intracorporeal Anastomosis

RRCEA = Robotic Right Colectomy Extracorporeal Anastomosis, LRCEA = Laparoscopic Right Colectomy Extracorporeal Anastomosis

This multi-institutional retrospective propensity score-matched analysis of minimally invasive laparoscopic and robotic-assisted right colectomies demonstrates that the intracorporeal anastomosis is associated with fewer conversions to open, shorter hospital LOS, and fewer overall complications after discharge when compared to minimally invasive laparoscopic and robotic-assisted right colectomies with an extracorporeal anastomosis. Though estimated blood loss and time to gastrointestinal recovery were statistically different, these differences were small and unlikely to be significantly different in the clinical setting. The number of individual postoperative complications prior to and after discharge were quite small (Table 4 and Table 5). Therefore, no statistical comparisons were provided for individual postoperative complications as definitive conclusions would be difficult to determine.

### Minimally invasive studies comparing intra- and extracorporeal anastomosis

Other studies including two meta-analyses also revealed short-term outcomes advantages with the laparoscopic IA approach, with some showing significantly shorter intestinal recovery time, less analgesic requirements, shorter hospital LOS, and less short-term morbidity when compared to the EA group.[14, 16] These authors suggest that the paucity of literature prior to these meta-analyses comparing IA and EA anastomoses was due to the technical challenges of laparoscopic stapling and suturing.[22, 25, 27–29]

Robotic-assisted studies to date have also shown short-term outcomes advantages to the IA approach including intestinal recovery time and hospital LOS.[15, 17] Previous robotic-assisted reports were comparisons of the robotic-assisted IA approach with the laparoscopic EA approach. These studies showed that the robotic-assisted intracorporeal group had significantly shorter hospital LOS than the laparoscopic extracorporeal group but there was no difference when compared to the laparoscopic intracorporeal group.[17] One study showed fewer anastomotic complications and incisional hernias in the robotic-assisted intracorporeal group when compared to the laparoscopic extracorporeal group.[15] Another study concluded that the intracorporeal anastomosis may facilitate extraction of longer specimens with less trauma through smaller incisions.[30] Other laparoscopic right colectomy studies have confirmed shorter incisions and better cosmetic results for the intracorporeal approach.[22, 24, 25, 28] Our study differs from prior published studies in that we performed a multi-center analysis with a larger sample size with results that may be more generalizable.

### Specific outcomes

#### Conversion and operative time

Our study revealed significantly fewer conversions and longer operating time for the IA group. Other studies reveal conflicting results with some showing significantly fewer conversions for the MIS intracorporeal approach when compared to the extracorporeal approach and others that show no significant difference.[21, 25, 31] Future studies that include subgroup analysis may suggest that patients with higher BMI may have fewer conversions if selected for the intracorporeal technique. Operative times are also inconclusive with some reports showing longer times for the IA approach and others showing no difference or shorter times when compared to EA.[22, 24, 25, 28, 31] Operative times may improve with experience and upon completion of the surgeon learning curve.[32, 33]

#### Intestinal recovery

Others have confirmed the outcomes advantages of the intracorporeal option for MIS right colectomy with respect to gastrointestinal recovery time.[15–17, 21–25] The entire operation including ileocolic mobilization, vessel ligation, takedown of the mesentery, division of the ileum and transverse colon, and anastomosis is done entirely prior to specimen extraction. With the intracorporeal anastomosis, there is no need to perform one of the most important parts of the operation through a small incision with poor visualization and there is no unintentional twisting or mesenteric stretching that can result in edema, bleeding, and resultant delayed intestinal recovery. The extracorporeal technique often requires more mobilization of a transverse colon that may not easily reach an extracorporeal extraction site incision.[14, 15, 17, 34–37] For some patients with high BMI, short/thick mesentery, and thick abdominal wall, the only minimally invasive option may be an intracorporeal anastomosis with less dissection of tissues that remain in situ and without the risk of mesenteric injury and need to lengthen the extraction site incision.[22] The advantages of the intracorporeal approach are for those capable of mastering minimally invasive suturing techniques. The robotic-assisted platform may be more suited for many surgeon skill sets due to the vision, articulating instruments, and other ergonomic advantages.[22, 31]

#### Hospital LOS

Several studies demonstrate an advantage for the intracorporeal anastomosis for hospital LOS while others show no significant difference between groups.[14–17, 21–25, 27, 28, 30, 31] This outcome is related to intestinal recovery time but there are other potentially confounding factors that contribute. We were unable to control for programs implementing Enhanced Recovery Pathways of varying degrees of standardization that decrease hospital LOS.[38, 39]

#### Postoperative complications

Most studies show no difference in postoperative complications between intra- and extracorporeal right colectomy groups. Our study showed a significant difference in postoperative complications after discharge up to 30-days. This significant difference was not apparent with respect to a specific complication but rather a cumulative effect of all postoperative complications. The difference in complications between groups has several possible explanations–extraction site difficulties in the extracorporeal group may be one. Though IA vs EA decision making was characterized by the method employed by the surgeon at the time of the study, it is possible that IA serves as a proxy for surgeons further along in their learning curve.

#### Incisional hernia and incision size

The right colectomy extraction site for the extracorporeal approach is typically the midline where the incisional hernia rate is highest.[16, 18–20] Several studies have shown that the MIS intracorporeal anastomosis allows specimen extraction at off-midline sites and the Pfannenstiel location with decreased risk for subsequent incisional hernias.[16, 18–20] Our multicenter retrospective study design made it difficult to obtain incisional length and hernia metrics and so we did not include these data points. Recognizing this limitation, this study serves as a reference for a prospective study that is currently underway with a focus on comparing incisional hernia rates for intracorporeal and extracorporeal techniques.

This study has inherent limitations of any retrospective study with respect to dissimilar comparison groups. There was no way to control for regional differences in multicenter patient populations, surgeon variations in techniques, and surgical decision-making when choosing an intracorporeal or extracorporeal anastomosis. To address this potential limitation, surgeons contributed patients to the group that defined the technique they were using at the time of the study. That is, surgeons did not choose one technique over the other based on operative degree of difficulty. We could not control for the degree of intracorporeal dissection prior to extracorporeal extraction. It is possible that more limited intracorporeal mobilization and dissection prior to extracorporeal extraction could negatively impact results for this group. This is also a potential strength of the study, however, recognizing that this comparison is not just about the anastomosis but a comparison of all segments of the operation including mobilization techniques, likely less standardized with respect to the extracorporeal approach. Though time to first bowel movement was significantly different in this study, the difference was small (2.7 ± 1.5 days vs 2.9 ± 1.5 days) and is likely not clinically significant. There may have been other unmeasured differences in techniques between clinical sites that could potentially impact results.

We used propensity matching to account for selection bias but even this method may not account for all potential confounders. However, in the absence of well-designed prospective comparative studies, propensity score matching from real world electronic medical record data provides a surrogate model to adjust for patient population heterogeneity. Some important variables like incisional hernias, incision size, and the concurrent implementation of Enhanced Recovery Pathways were not able to be collected for this study and will be the subject of our prospective study. Nevertheless, this study is unique in that it brings together both MIS options in both study groups for comparison and provides generalizable data.

The traditional open approach to right colectomy is still common with MIS techniques adopted in only 50–60% of cases.[9–13] There is a need to increase MIS training efforts and options. The intracorporeal anastomosis is an advantage for both MIS approaches.[22, 24, 25, 28, 31] Operative times may improve with experience and upon completion of the learning curve.[22, 24, 25, 28, 31–33] This study that presents laparoscopic and robotic-assisted data together in both study arms may help guide MIS choices for open and extracorporeal surgeons who recognize the value of the intracorporeal anastomosis.

## Conclusions

This multi-institutional propensity score comparison of minimally invasive laparoscopic and robotic-assisted intracorporeal and extracorporeal anastomosis after right colectomy demonstrates several short-term outcomes advantages for the intracorporeal approach. These data may guide surgeons focused on upgrading minimally invasive training efforts and those choosing minimally invasive options for colectomies.

## References

[1] Nelson H, Sargent DJ, Wieand HS, Fleshman J, Anvari M, Stryker SJ, et al A comparison of laparoscopically assisted and open colectomy for colon cancer. N Engl J Med. 2004;350(20):2050–9. 10.1056/NEJMoa032651 15141043

[2] Bhama AR, Obias V, Welch KB, Vandewarker JF, Cleary RK. A comparison of laparoscopic and robotic colorectal surgery outcomes using the American College of Surgeons National Surgical Quality Improvement Program (ACS NSQIP) database. Surg Endosc. 2016;30(4):1576–84. 10.1007/s00464-015-4381-9 26169638

[3] Bonjer HJ, Deijen CL, Abis GA, Cuesta MA, van der Pas MH, de Lange-de Klerk ES, et al A randomized trial of laparoscopic versus open surgery for rectal cancer. N Engl J Med. 2015;372(14):1324–32. 10.1056/NEJMoa1414882 25830422

[4] Guillou PJ, Quirke P, Thorpe H, Walker J, Jayne DG, Smith AM, et al Short-term endpoints of conventional versus laparoscopic-assisted surgery in patients with colorectal cancer (MRC CLASICC trial): multicentre, randomised controlled trial. Lancet. 2005;365(9472):1718–26. 10.1016/S0140-6736(05)66545-2 15894098

[5] Hollis RH, Cannon JA, Singletary BA, Korb ML, Hawn MT, Heslin MJ. Understanding the value of both laparoscopic and robotic approaches compared to the open approach in colorectal surgery. J Laparoendosc Adv Surg Tech A. 2016;26(11):850–6. 10.1089/lap.2015.0620 27398733

[6] Kang CY, Chaudhry OO, Halabi WJ, Nguyen V, Carmichael JC, Stamos MJ, et al Outcomes of laparoscopic colorectal surgery: data from the Nationwide Inpatient Sample 2009. Am J Surg 2012;204(6):952–7. 10.1016/j.amjsurg.2012.07.031 23122910

[7] Tam MS, Kaoutzanis C, Mullard AJ, Regenbogen SE, Franz MG, Hendren S, et al A population-based study comparing laparoscopic and robotic outcomes in colorectal surgery. Surg Endosc. 2016;30(2):455–63. 10.1007/s00464-015-4218-6 25894448

[8] Yeo HL, Isaacs AJ, Abelson JS, Milsom JW, Sedrakyan A. Comparison of open, laparoscopic, and robotic colectomies using a large national database: outcomes and trends related to surgery center volume. Dis Colon Rectum. 2016;59(6):535–42. 10.1097/DCR.0000000000000580 27145311

[9] Damle RN, Macomber CW, Flahive JM, Davids JS, Sweeney WB, Sturrock PR, et al Surgeon volume and elective resection for colon cancer: an analysis of outcomes and use of laparoscopy. J Am Coll Surg. 2014;218(6):1223–30. 10.1016/j.jamcollsurg.2014.01.057 24768291 PMC4467094

[10] Halabi WJ, Kang CY, Jafari MD, Nguyen VQ, Carmichael JC, Mills S, et al Robotic-assisted colorectal surgery in the United States: a nationwide analysis of trends and outcomes. World J Surg. 2013;37(12):2782–90. 10.1007/s00268-013-2024-7 23564216

[11] Keller DS, Senagore AJ, Lawrence JK, Champagne BJ, Delaney CP. Comparative effectiveness of laparoscopic versus robot-assisted colorectal resection. Surg Endosc. 2014;28(1):212–21. 10.1007/s00464-013-3163-5 23996335

[12] Moghadamyeghaneh Z, Carmichael JC, Mills S, Pigazzi A, Nguyen NT, Stamos MJ. Variations in laparoscopic colectomy utilization in the United States. Dis Colon Rectum. 2015;58(10):950–6. 10.1097/DCR.0000000000000448 26347967

[13] Rea JD, Cone MM, Diggs BS, Deveney KE, Lu KC, Herzig DO. Utilization of laparoscopic colectomy in the United States before and after the clinical outcomes of surgical therapy study group trial. Ann Surg. 2011;254(2):281–8. 10.1097/SLA.0b013e3182251aa3 21685791

[14] Feroci F, Lenzi E, Garzi A, Vannucchi A, Cantafio S, Scatizzi M. Intracorporeal versus extracorporeal anastomosis after laparoscopic right hemicolectomy for cancer: a systematic review and meta-analysis. Int J Colorectal Dis. 2013;28(9):1177–86. 10.1007/s00384-013-1651-7 23371336

[15] Morpurgo E, Contardo T, Molaro R, Zerbinati A, Orsini C, D'Annibale A. Robotic-assisted intracorporeal anastomosis versus extracorporeal anastomosis in laparoscopic right hemicolectomy for cancer: a case control study. J Laparoendosc Adv Surg Tech A. 2013;23(5):414–7. 10.1089/lap.2012.0404 23627922

[16] Ricci C, Casadei R, Alagna V, Zani E, Taffurelli G, Pacilio CA, et al A critical and comprehensive systematic review and meta-analysis of studies comparing intracorporeal and extracorporeal anastomosis in laparoscopic right hemicolectomy. Langenbecks Arch Surg. 2017;402(3):417–27. 10.1007/s00423-016-1509-x 27595589

[17] Trastulli S, Coratti A, Guarino S, Piagnerelli R, Annecchiarico M, Coratti F, et al Robotic right colectomy with intracorporeal anastomosis compared with laparoscopic right colectomy with extracorporeal and intracorporeal anastomosis: a retrospective multicentre study. Surg Endosc. 2015;29(6):1512–21. 10.1007/s00464-014-3835-9 25303905

[18] Harr JN, Juo YY, Luka S, Agarwal S, Brody F, Obias V. Incisional and port-site hernias following robotic colorectal surgery. Surg Endosc. 2016;30(8):3505–10. 10.1007/s00464-015-4639-2 26541723

[19] Samia H, Lawrence J, Nobel T, Stein S, Champagne BJ, Delaney CP. Extraction site location and incisional hernias after laparoscopic colorectal surgery: should we be avoiding the midline? Am J Surg. 2013;205(3):264–7; discussion 8. 10.1016/j.amjsurg.2013.01.006 23375702

[20] Widmar M, Keskin M, Beltran P, Nash GM, Guillem JG, Temple LK, et al Incisional hernias after laparoscopic and robotic right colectomy. Hernia. 2016;20(5):723–8. 10.1007/s10029-016-1518-2 27469592 PMC5025379

[21] Arredondo Chaves J, Pastor Idoate C, Baixauli Fons J, Bellver Oliver M, Pedano Rodríguez N, Bueno Delgado Á, et al A case-control study of extracorporeal versus intracorporeal anastomosis in patients subjected to right laparoscopic hemicolectomy. Cirugía Española (English Edition). 2011;89(1):24–30. 10.1016/j.ciresp.2010.10.003 21176829

[22] Fabozzi M, Allieta R, Brachet Contul R, Grivon M, Millo P, Lale-Murix E, et al Comparison of short- and medium-term results between laparoscopically assisted and totally laparoscopic right hemicolectomy: a case-control study. Surg Endosc. 2010;24(9):2085–91. 10.1007/s00464-010-0902-8 20174945

[23] Magistro C, Lernia SD, Ferrari G, Zullino A, Mazzola M, De Martini P, et al Totally laparoscopic versus laparoscopic-assisted right colectomy for colon cancer: is there any advantage in short-term outcomes? A prospective comparative assessment in our center. Surg Endosc. 2013;27(7):2613–8. 10.1007/s00464-013-2799-5 23397503

[24] Roscio F, Bertoglio C, De Luca A, Frattini P, Scandroglio I. Totally laparoscopic versus laparoscopic assisted right colectomy for cancer. Int J Surg. 2012;10(6):290–5. 10.1016/j.ijsu.2012.04.020 22564829

[25] Scatizzi M, Kroning KC, Borrelli A, Andan G, Lenzi E, Feroci F. Extracorporeal versus intracorporeal anastomosis after laparoscopic right colectomy for cancer: a case-control study. World J Surg. 2010;34(12):2902–8. 10.1007/s00268-010-0743-6 20703468

[26] Faries D, Obenchain RL, Leon AC, Haro JM. Analysis of observational health care data using SAS Cary, NC: SAS Institute; 2010.

[27] Grams J, Tong W, Greenstein AJ, Salky B. Comparison of intracorporeal versus extracorporeal anastomosis in laparoscopic-assisted hemicolectomy. Surg Endosc. 2010;24(8):1886–91. 10.1007/s00464-009-0865-9 20112118

[28] Hellan M, Anderson C, Pigazzi A. Extracorporeal versus intracorporeal anastomosis for laparoscopic right hemicolectomy. JSLS. 2009;13(3):312–7. 19793468 PMC3015972

[29] van Oostendorp S, Elfrink A, Borstlap W, Schoonmade L, Sietses C, Meijerink J, et al Intracorporeal versus extracorporeal anastomosis in right hemicolectomy: a systematic review and meta-analysis. Surg Endosc. 2017;31(1):64–77. 10.1007/s00464-016-4982-y 27287905 PMC5216072

[30] Lujan HJ, Plasencia G, Rivera BX, Molano A, Fagenson A, Jane LA, et al Advantages of robotic right colectomy with intracorporeal anastomosis. Surg Laparosc Endosc Percutan Tech. 2018;28(1):36–41. 10.1097/SLE.0000000000000384 28319493 PMC5802257

[31] Hanna MH, Hwang GS, Phelan MJ, Bui TL, Carmichael JC, Mills SD, et al Laparoscopic right hemicolectomy: short- and long-term outcomes of intracorporeal versus extracorporeal anastomosis. Surg Endosc. 2016;30(9):3933–42. 10.1007/s00464-015-4704-x 26715015

[32] de'Angelis N, Lizzi V, Azoulay D, Brunetti F. Robotic versus laparoscopic right colectomy for colon cancer: analysis of the initial simultaneous learning curve of a surgical fellow. J Laparoendosc Adv Surg Tech A. 2016;26(11):882–92. 10.1089/lap.2016.0321 27454105

[33] Parisi A, Scrucca L, Desiderio J, Gemini A, Guarino S, Ricci F, et al Robotic right hemicolectomy: analysis of 108 consecutive procedures and multidimensional assessment of the learning curve. Surg Oncol. 2017;26(1):28–36. 10.1016/j.suronc.2016.12.005 28317582

[34] Abraham NS, Young JM, Solomon MJ. Meta-analysis of short-term outcomes after laparoscopic resection for colorectal cancer. Br J Surg. 2004;91(9):1111–24. 10.1002/bjs.4640 15449261

[35] Cirocchi R, Trastulli S, Farinella E, Guarino S, Desiderio J, Boselli C, et al Intracorporeal versus extracorporeal anastomosis during laparoscopic right hemicolectomy—systematic review and meta-analysis. Surg Oncol. 2013;22(1):1–13. 10.1016/j.suronc.2012.09.002 23116767

[36] Senagore AJ, Delaney CP, Brady KM, Fazio VW. Standardized approach to laparoscopic right colectomy: outcomes in 70 consecutive cases. J Am Coll Surg. 2004;199(5):675–9. 10.1016/j.jamcollsurg.2004.06.021 15501105

[37] Tarta C, Bishawi M, Bergamaschi R. Intracorporeal ileocolic anastomosis: a review. Tech Coloproctol. 2013;17(5):479–85. 10.1007/s10151-013-0998-7 23519986

[38] Martin TD, Lorenz T, Ferraro J, Chagin K, Lampman RM, Emery KL, et al Newly implemented enhanced recovery pathway positively impacts hospital length of stay. Surg Endosc. 2016;30(9):4019–28. 10.1007/s00464-015-4714-8 26694181

[39] Miller PE, Dao H, Paluvoi N, Bailey M, Margolin D, Shah N, et al Comparison of 30-day postoperative outcomes after laparoscopic vs robotic colectomy. J Am Coll Surg. 2016;223(2):369–73. 10.1016/j.jamcollsurg.2016.03.041 27109780

